# SPARC Is a Key Regulator of Proliferation, Apoptosis and Invasion in Human Ovarian Cancer

**DOI:** 10.1371/journal.pone.0042413

**Published:** 2012-08-03

**Authors:** Jie Chen, Mei Wang, Bo Xi, Jian Xue, Dan He, Jie Zhang, Yueran Zhao

**Affiliations:** 1 Department of Maternal and Child Health Care, School of Public Health, Shandong University, Jinan, China; 2 Pharmacy Department, Shandong Provincial Hospital affiliated to Shandong University, Jinan, China; 3 Central Laboratory, Shandong Provincial Hospital affiliated to Shandong University, Jinan, China; Florida International University, United States of America

## Abstract

**Background:**

Secreted protein acidic and rich in cysteine (SPARC), a calcium-binding matricellular glycoprotein, is implicated in the progression of many cancers. In this study, we investigated the expression and function of SPARC in ovarian cancer.

**Methods:**

cDNA microarray analysis was performed to compare gene expression profiles of the highly invasive and the low invasive subclones derived from the SKOV3 human ovarian cancer cell line. Immunohistochemistry (IHC) staining was performed to investigate SPARC expression in a total of 140 ovarian tissue specimens. In functional assays, effects of SPARC knockdown on the biological behavior of ovarian cancer cells were investigated. The mechanisms of SPARC in ovarian cancer proliferation, apoptosis and invasion were also researched.

**Results:**

SPARC was overexpressed in the highly invasive subclone compared with the low invasive subclone. High SPARC expression was associated with high stage, low differentiation, lymph node metastasis and poor prognosis of ovarian cancer. Knockdown of SPARC expression significantly suppressed ovarian cancer cell proliferation, induced cell apoptosis and inhibited cell invasion and metastasis.

**Conclusion:**

SPARC is overexpressed in highly invasive subclone and ovarian cancer tissues and plays an important role in ovarian cancer growth, apoptosis and metastasis.

## Introduction

Ovarian cancer is the second most common gynecologic cancer and one of the leading causes of cancer deaths in women [1]. High percentage of ovarian cancer patients are diagnosed at an advanced stage. Although substantial advances have been made in ovarian cancer research, survival to incidence ratio is still poor and overall cure rate remains very low [2]. Tumor recurrence and metastasis are considered the major reasons for poor clinical outcome and cancer deaths [3]. Therefore, studying the mechanism of tumor invasion and metastasis will provide further insights into the development and progression of ovarian cancer.

SPARC (secreted protein acidic and rich in cysteine), also termed osteonectin, BM-40, and 43 K protein, is a calcium-binding matricellular glycoprotein, whose function is to modulate cell–matrix interactions and cell function without participating in the structural scaffold of the extracellular matrix [4]. Although there is growing evidence for an important role for SPARC in a variety of cancers, there is no unifying model, which explains all facets of its function and contribution to the development and progression of cancer [5]. SPARC is differentially expressed in tumors and its surrounding stroma in various cancers in comparison to the normal tissue. For example, higher levels of SPARC expression have been reported in breast cancer [6], [7], hepatocellular carcinoma [8], [9], prostate cancer [10], colorectal cancer [11], [12], and ovarian cancer [13], [14]. However, an opposite correlation has also been demonstrated, suggesting that SPARC may be able to inhibit tumorigenesis or tumor progression in breast cancer [15], [16], hepatocellular carcinoma [17], prostate cancer [18], colorectal cancer [19], [20], and ovarian cancer [21]. Therefore, the function of SPARC in cancer merits further investigation.

In the present study, we performed cDNA microarray analysis to investigate the differential gene expression profile of the highly invasive subclone S1 and the low invasive subclone S21, both of which were derived from the SKOV3 human ovarian cancer cell line. We found that many genes were differentially expressed in these two types of subclones. Particularly, SPARC was found to be significantly overexpressed in the highly invasive subclone S1 compared with that in the low invasive subclone S21. To clarify the relationship between SPARC and ovarian cancer progression, the expressions of SPARC in human ovarian tissue specimens were measured by immunohistochemistry (IHC). In function assay, by lentivirus-mediated RNA interference, we decreased the expression of SPARC in highly invasive subclone S1 and HO8910PM to determine the effect of SPARC on ovarian cancer cell proliferation, apoptosis, invasion and metastasis.

## Materials and Methods

### Cell Lines

SKOV3, NIH3T3 and HO8910PM (a highly metastatic ovarian cancer cell line [22]) cell lines were obtained from Shanghai Institute for Biological Sciences, Chinese Academy of Sciences. The highly invasive subclone (S1) and the low invasive subclone (S21) were derived from the SKOV3 human ovarian cancer cell line [23]. Cells were cultured in RPMI-1640 for SKOV3 or DMEM for NIH3T3 and HO8910PM supplemented with 10% fetal bovine serum (FBS) and antibiotics (Gibco BRL, Rockville, MD).

**Table 1:** Real time RT-PCR primers.

Genes	Forward and reverse primer	Productlength (bp)
SPARC	F: 5′-ACATAAGCCCAGTTCATCACCA-3′	278
	R: 5′-ACAACCGATTCACCAACTCCA-3′	
E-cadherin	F: 5′- GGATTGCAAATTCCTGCCATTC -3′	147
	R: 5′- AACGTTGTCCCGGGTGTCA -3′	
β-catenin	F: 5′-GCTGATCTTGGACTTGATATTGGTG -3′	117
	R: 5′- GTCCATACCCAAGGCATCCTG -3′	
α-catenin	F: 5′- CTCTACTGCCACCAGCTGAACATC -3′	154
	R: 5′- ATGCCTTCACTGTCTGCACCAC -3′	
Integrin β1	F: 5′-CAAGCAGGGCCAAATTGTGG-3′	185
	R: 5′-CCTTTGCTACGGTTGGTTACATT-3′	
Integrin β3	F: 5′-TTCAATGCCACCTGCCTCAA-3′	98
	R: 5′-TTGGCCTCAATGCTGAAGCTC-3′	
P53	F: 5′- AACGGTACTCCGCCACC-3′	94
	R: 5′- CGTGTCACCGTCGTGGA-3′	
P21	F: 5′- CACTCAGAGGAGGAAAATCCAGT -3′	90
	R: 5′- TTCTGACATGGCGCCTGCCT -3′	
Cyclin D1	F: 5′-CCGAGAAGCTGTGCATCTACAC-3′	94
	R: 5′-AGGTTCCACTTGAGCTTGTTCAC-3′	
PCNA	F: 5′- CTGTAGCGGCGTTGT -3′	133
	R: 5′- ACTTTCTCCTGGTTTGG -3′	
Bcl-2	F: 5′- TCAGGGACGGGGTGAACT -3′	143
	R: 5′- CAGGTGCCGGTTCAGGTACTC -3′	
Bax	F: 5′- CGCCGTGGACACAGACTC -3′	108
	R: 5′- GCAAAGTAGAAAAGGGCGACAAC -3′	
u-PA	F: 5′-TCTGCCTGCCCTCGATGTATAAC-3′	179
	R: 5′-GGTGGTGACTTCAGAGCCGTAGTAG-3′	
PAI-1	F: 5′-GGTCTCCAAACCAGACGGTGA-3′	188
	R: 5′-TGGCAATGTGACTGGAACAGAAATA-3′	
uPAR	F: 5′- ATCACCAGCCTTACCGAGGTTG -3′	87
	R: 5′- ACGGCTTCGGGAATAGGTGAC -3′	
MMP2	F: 5′-TGACATCAAGGGCATTCAGGAG-3′	134
	R: 5′-TCTGAGCGATGCCATCAAATACA-3′	
MMP9	F: 5′- CGCCCATTTCGACGATGAC -3′	80
	R: 5′- CGCCATCTGCGTTTCCAA -3′	
TIMP1	F: 5′- ACAGACGGCCTTCTGCAATTC-3′	166
	R: 5′- GGTGTAGACGAACCGGATGTCA -3′	
TIMP2	F: 5′- GTTCAAAGGGCCTGAGAAGGA -3′	166
	R: 5′- CCAGGGCACGATGAAGTCA-3′	
β-actin	F: 5′-CCACGAAACTACCTTCAACTCCA-3′	131
	R: 5′-GTGATCTCCTTCTGCATCCTGTC-3′	

### Microarray Analysis

Total RNA was extracted from the highly invasive subclone (S1) and the low invasive subclone (S21) using RNeasy Mini kit (Qiagen, Valencia, CA). RNA quality was assessed by spectrophotometry and denaturing gel electrophoresis. RNA was amplified and labeled using Agilent Quick Amp labeling kit and hybridized to Agilent whole genome oligo microarray. Slides were scanned using Agilent DNA microarray scanner. Data were processed using Agilent Feature Extraction Software (version 10.5.1.1) and analyzed using Agilent GeneSpring GX software (version 11.0). The experiments were performed in triplicate.

### Tissue Specimens

Tissue specimens were obtained with the written informed consent from 80 women with epithelial ovarian cancer (with 29 serous cystadenocarcinoma, 25 mucinous cystadenocarcinoma and 26 endometrioid carcinoma), from 35 women with benign ovarian tumor, and from 25 normal control ovary tissue at the Department of Gynecology and Obstetrics, Shandong Provincial Hospital between 2005 and 2007. All of the ovarian cancer patients were clinically staged according to the FIGO staging system [with 38 low stage tumors (FIGO stages I and II) and 42 high stage tumors (FIGO stages III and IV)]. None of the ovarian cancer patients received preoperative radiation or chemotherapy. The study was approved by the Institutional Medical Ethics Committee of Shandong University.

### Immunohistochemistry (IHC)

According to standard streptavidin-biotin-peroxidase complex procedures, IHC was performed on formalin-fixed, paraffin-embedded sections (5 µm thick) and cell slides fixed in 4% paraformaldehyde. Briefly, after dewaxing, rehydration, and antigen retrieval, the sections were incubated with anti-human SPARC antibody (AF941, R&D Systems and bs-1133R, BIOSS) with working dilution 15 µg/ml for AF941 and 10 µg/ml for bs-1133R at 4°C overnight. The secondary antibody was horseradish peroxidase conjugated anti-goat IgG for AF941 and anti-rabbit IgG for bs-1133R. A negative control was obtained by replacing the primary antibody with normal rabbit immunoglobulin (IgG). Positive expression of SPARC protein was defined as the presence of brown granules in the cytoplasm or stroma.

**Figure 1:**
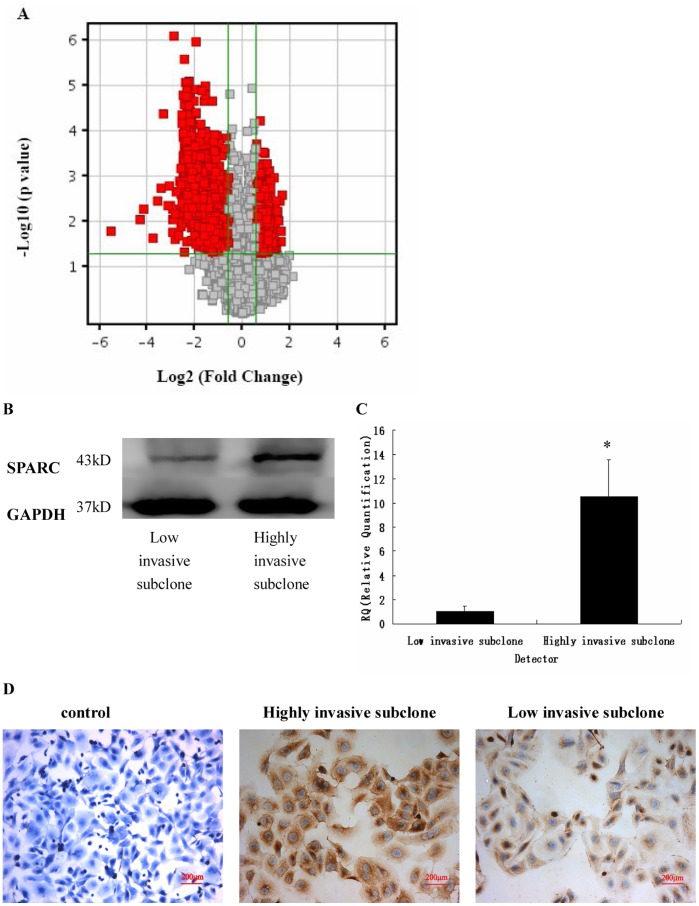
SPARC expression in the highly invasive subclone S1 and the low invasive subclone S21. (A) Scatter plot showing fold-change values versus P-values for visualizing differential expression between the highly invasive and low invasive clones. The vertical lines represent 1.5-fold up- and down-regulation, respectively. The horizontal line represents a P-value of 0.05. The red points in the plot represent the differentially expressed genes with statistical significance. (B, C, D) SPARC expression as measured by Western blot (B), q-RT-PCR (C), and immunohistochemistry (D) (Magnification ×200). *P<0.05.

### Immunohistochemistry (IHC) Analysis

A semiquantitative scoring system based on intensity of staining and distribution of positive cells was used to evaluate SPARC expression [24]. The intensity of SPARC positive staining ranged from 0 to 3 (negative = 0, weak = 1, moderate = 2, or strong = 3) and the percentage of positively stained cells was scored as 0 (0%), 1 (1 to 25%), 2 (26 to 50%), 3 (51 to 75%), and 4 (76 to 100%). The sum of the intensity and percentage score was used as the final staining scores (0 to 7). The sum-indexes (−), (+), (++), and (+++) indicated final staining score of 0, 1–3, 4–5, and 6–7, respectively. For statistical analysis, sum-indexes (−) and (+) were defined as low SPARC expression, while sum-indexes (++) and (+++) were defined as high SPARC expression. Each section was independently scored by two pathologists. If an inconsistency occurred, a third pathologist was consulted to achieve consensus.

### Peptide Blocking Assay

In order to test the specificity of anti-SPARC, the antibody was incubated overnight at 4°C with recombinant Human SPARC (941-SP, R&D Systems). Immunohistochemistry was then performed as described above, but the blocked anti-SPARC was used as primary antibody.

**Figure 2:**
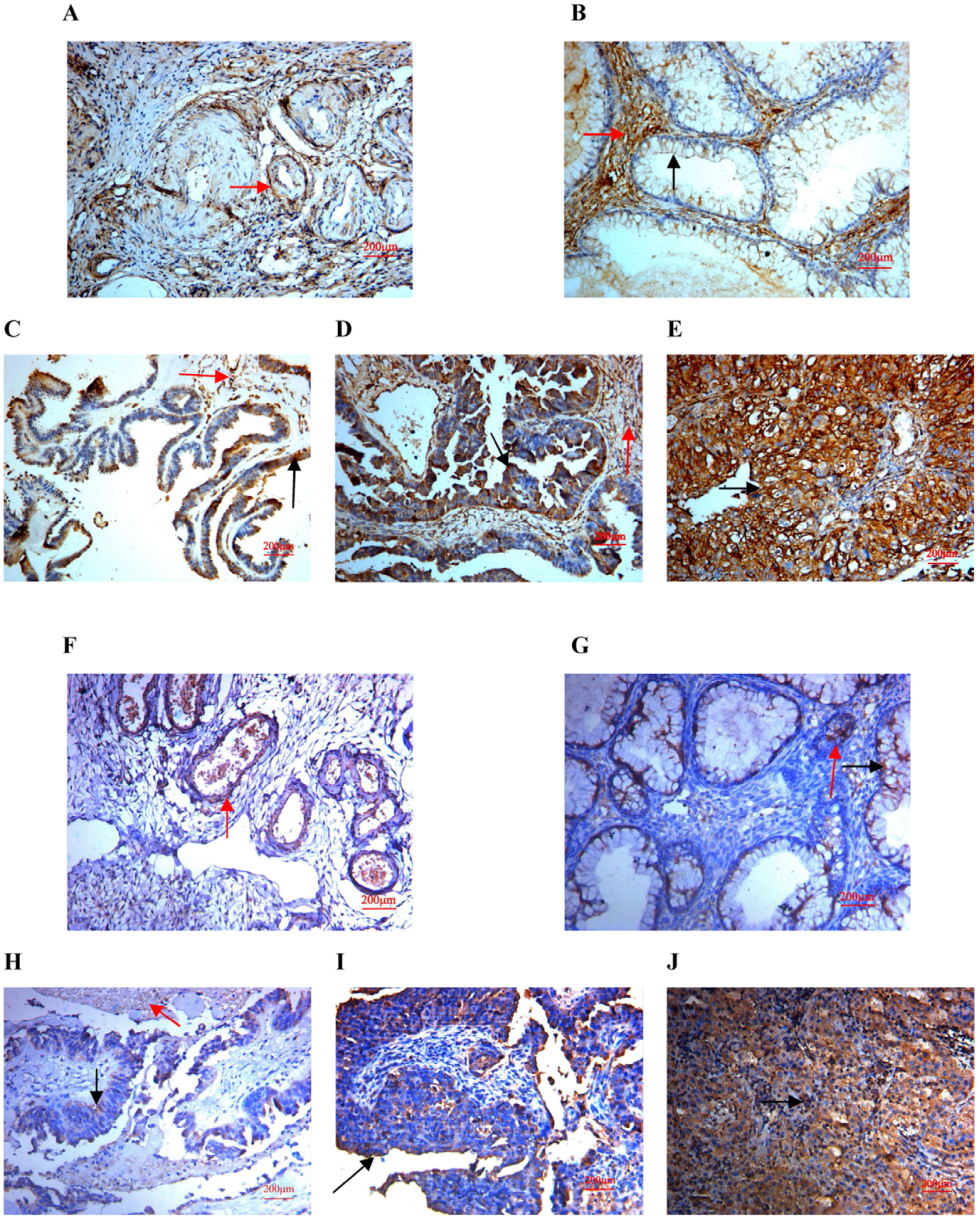
Expression of SPARC in human ovarian tissues using antibody AF941 and bs-1133R. (A) Normal human ovarian tissue using antibody AF941, (B) Benign ovarian tumor using antibody AF941, (C) High differentiation of ovarian carcinoma using antibody AF941, (D) Medium differentiation of ovarian carcinoma using antibody AF941, (E) Low differentiation of ovarian carcinoma using antibody AF941, (F) Normal human ovarian tissue using antibody bs-1133R, (G) Benign ovarian tumor using antibody bs-1133R, (H) High differentiation of ovarian carcinoma using antibody bs-1133R, (I) Medium differentiation of ovarian carcinoma using antibody bs-1133R, (J) Low differentiation of ovarian carcinoma using antibody bs-1133R (Magnification ×200). Black arrows indicate cell cytoplasm stained, red arrows indicate stroma stained.

### Real-time Quantitative RT-PCR (q-RT-PCR)

Total RNA was extracted using Trizol reagent (Invitrogen) and reversed transcribed. Quantitative real-time PCR analysis was performed using ABI PRISM 7500 Real-Time PCR System (Applied Biosystems). Each well (20 µl reaction volume) contained 10 µl Power SYBR Green PCR master mix (Applied Biosystems), 1 µl of each primer (5 µmol/l) and 1µl cDNA template (50 ng/µl). The primers (table 1) were designed by primer 5 software and synthesized by TaKaRa Biotechnology (Dalian) Co., Ltd.

### Western Blot

Cells were lysed in RIPA buffer containing 1mM PMSF. Fifty microgram of protein per lane was resolved by SDS-PAGE and transferred to PVDF membrane and blocked with 5% BSA. The membranes were first incubated with primary antibody against SPARC, PCNA, cyclin D1, P53, P21, Bax and Bcl-2 at 1∶1000 dilutions overnight, and then incubated with secondary antibody horseradish peroxidase-conjugated anti-goat or anti-mouse IgG for 1 hour at room temperature, blots were developed using ECL method. Band intensity was analyzed using Gel-Pro Analyzer Software (Media Cybernetics, Inc., Bethesda, MD).

### RNA Interference

Self-inactivating lentivirus vector (GeneChem, Shanghai, China) containing a CMV-driven GFP reporter and a U6 promoter upstream of the cloning sites (Age I and EcoR I) was used for cloning small hairpin RNAs (shRNAs). The target sequence for SPARC was 5′- AACAAGACCTTCGACTCTTCC-3′; the negative control sequence was 5′-TTCTCCGAACGTGTCACGT-3′. The highly invasive subclone cells (S1 and HO8910PM) were cultured in six-well tissue culture plates and infected with lentivirus at a multiplicity of infection (MOI) of 100 for 24 h. Then the medium was replaced with fresh complete medium. After 4 days, cells were observed under fluorescence microscopy to confirm that more than 80% of cells were GFP-positive.

**Table 2:** Expression of SPARC in human ovarian tissues with AF941 antibody.

	N	SPARC low		SPARC high		*X^2^*	*P*
		(−/+)		(++/+++)			
		n	%	n	%		
Normal	25	21	84	4	16	27.96	8.49×10^−7^
Benign	35	22	62.9	13	37.5		
Carcinoma	80	23	28.8	57	71.2		
Pathology type						0.12	0.94
*serous cystadenocarcinoma*	29	9	31.0	20	69.0		
*mucinous cystadenocarcinoma*	25	7	28.0	18	72.0		
*endometrioid carcinoma*	26	7	26.9	19	73.1		
Cell differentiation						10.14	6.28×10^−3^
* High*	27	13	48.1	14	51.9		
* Medium*	23	7	30.4	16	70.6		
* Low*	30	3	10	27	90		
Tumor stage						14.33	1.53×10^−4^
* Low stage*	38	19	50.0	19	50.0		
* High stage*	42	4	9.5	38	90.5		
Nodal status						10.27	1.35×10^−3^
* Positive*	43	6	13.9	37	86.1		
* Negative*	37	17	45.9	20	54.1		

**Table 3:** Expression of SPARC in human ovarian tissues with bs-1133R antibody.

	N	SPARC low		SPARC high		*X^2^*	*P*
		(−/+)		(++/+++)			
		n	%	n	%		
Normal	25	22	88	3	12	31.90	1.63×10^−8^
Benign	35	20	57.1	15	42.9		
Carcinoma	80	21	26.3	59	73.7		
Pathology type						0.004	0.948
*serous cystadenocarcinoma*	29	8	27.6	21	72.4		
*mucinous cystadenocarcinoma*	25	6	24.0	19	76.0		
*endometrioid carcinoma*	26	7	26.9	19	73.1		
Cell differentiation						10.43	0.001
* High*	27	12	44.4	15	55.6		
* Medium*	23	7	30.4	16	70.6		
* Low*	30	2	6.7	28	93.3		
Tumor stage						16.67	4.44×10^−5^
* Low stage*	38	18	47.4	20	52.6		
* High stage*	42	3	7.1	39	92.9		
Nodal status						10.27	0.001
* Positive*	43	5	11.6	38	88.4		
* Negative*	37	16	43.2	21	56.8		

### Cell Proliferation Assays

Cell proliferation was determined by using MTT assay and anchorage independent soft agar colony formation assay. For MTT assay, 20 µl MTT (5 mg/ml in PBS) was added directly into each well of 96-well plate and incubated at 37°C for 4 h. Then media was removed and 200 µl DMSO was added to dissolve formazan crystals. The optical density at 570 nm was read with a microplate reader (Molecular Devices, Sunnyvale, CA).

For soft agar colony formation assay, 500 µl 2×DMEM supplemented with 20% FBS was mixed with 500 µl 1.2% Sea Plague agar and solidified in each well of a 24-well plate to form base agar layer. For top agar layer, 25 µl cells (5×10^3^/ml) were mixed with 500 µl 2×DMEM and 500 µl 0.7% Sea Plague agar and added on top of base agar layer. After grown for 14 days, colony formation was monitored under microscopy. A cluster of ten cells or more was defined as a colony.

### Annexin V-PI Assays for Apoptosis

Cells were collected and washed twice with PBS, suspended in 200 µL binding buffer and 10 µL Annexin V-FITC for 20 minutes in the dark, and thereafter, 300 µL binding buffer and 5 µL propidium iodide (PI) were added to each sample. The apoptotic cells were determined using a flow cytometer (Becton Dickinson) with CellQuest (Becton Dickinson) software.

### Analysis of Cell Cycle Distribution

Cells were harvested, washed with ice-cold PBS, and stained with 50 µg/mL PI and 250 µg/mL RNase for 30 minutes. The percentage of cells in each phase of the cell cycle was determined with a computer-programmed ModFit LT2.0 DNA assay (Becton Dickinson) using a flow cytometer.

### Cell Invasion Assay and Migration Assay

Invasion assay was performed as described previously [25]. Each Transwell (BD Biosciences, Bedford, MA) was coated with 50 µl 1∶3 dilution of Matrigel (BD Biosciences, Bedford, MA). Cells (0.2×10^6^) were resuspended in 200 µl serum-free media and seeded into the upper chamber. Conditioned media of NIH3T3 cell culture was filtered and added to the lower chamber as a chemotactic factor. After 12 h, non-invading cells remaining on the upper surface were removed, and cells on the lower surface were fixed, stained with hematoxylin and eosin (H&E), and counted. Cell migration assay was also performed using Transwells without Matrigel coating. Each experiment was performed at least in triplicate. Invading and migration cell numbers of Boyden chambers were counted and the data were expressed as mean ± SE.

**Figure 3:**
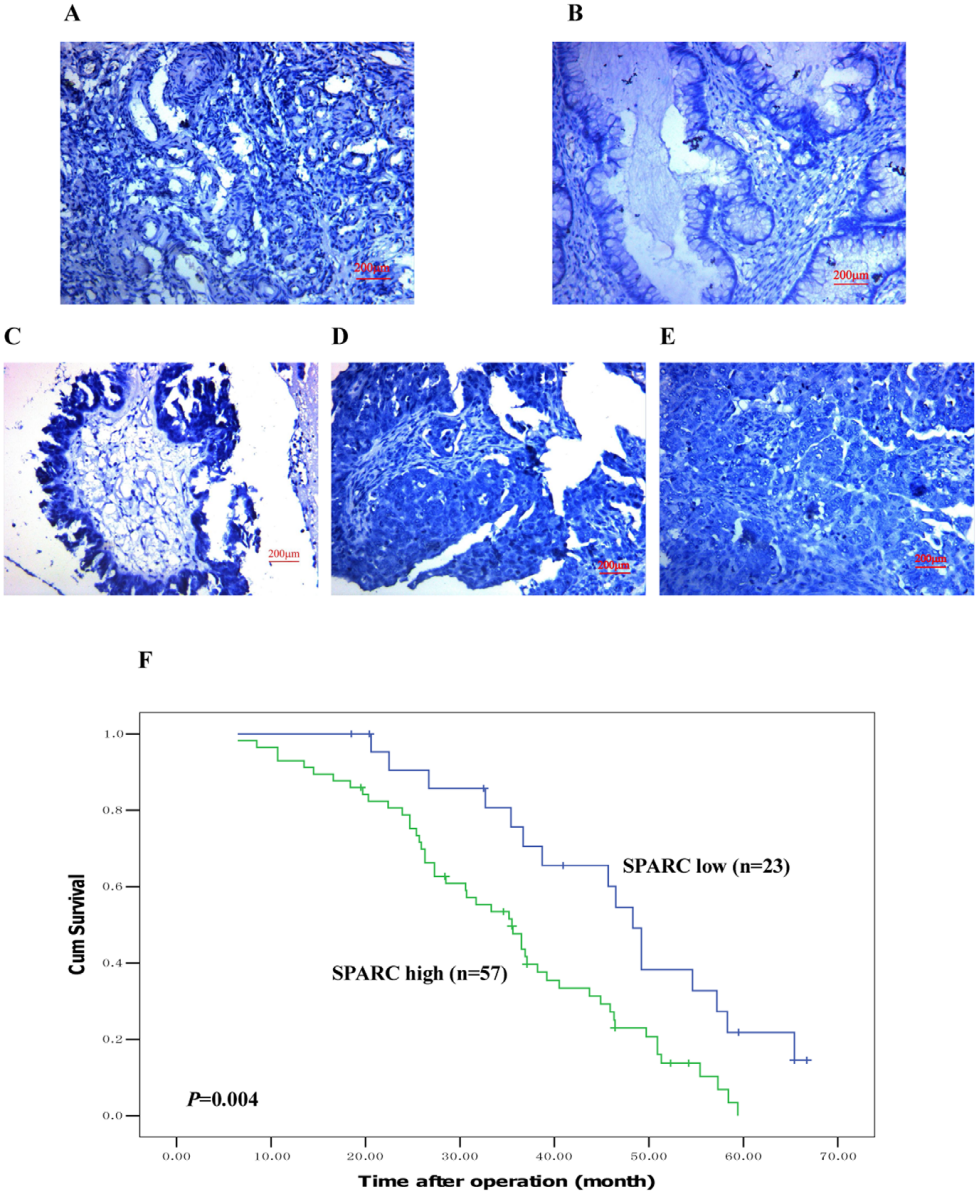
Peptide blocking assay with recombinant Human SPARC. (A) Normal human ovarian tissue, (B) Benign ovarian tumor, (C) High differentiation of ovarian carcinoma, (D) Medium differentiation of ovarian carcinoma, (E) Low differentiation of ovarian carcinoma (Magnification ×200). (F) Kaplan-Meier analysis about the overall survival of the patients whose tumors had high or low SPARC expression.

### Tumor Xenografts in Nude Mice

BALB/C-nu/nu 5-week-old female nude mice were purchased from National Resource Center for Rodent Laboratory Animal of China. Five nude mice were injected subcutaneously with 5.0×10^6^ cells, and ten nude mice were injected through the tail veins with the same cells once a week for 3 consecutive weeks. The mice were maintained in a sterile animal facility and monitored for tumor growth. The volumes of tumors were monitored at the indicated times and calculated according to the formula: 0.5×length×width^2^. After 3 months, the mice were killed and the tumor and lung were dissected and examined histologically. Paraffin sections of the lung tissues were made, stained with hematoxylin and eosin (H&E) and observed under a microscope. The average values were expressed as mean ± SE. This animal experiment was approved by the Institutional Animal Care and Use Committee of Shandong University and was in compliance with all regulatory guidelines.

**Figure 4:**
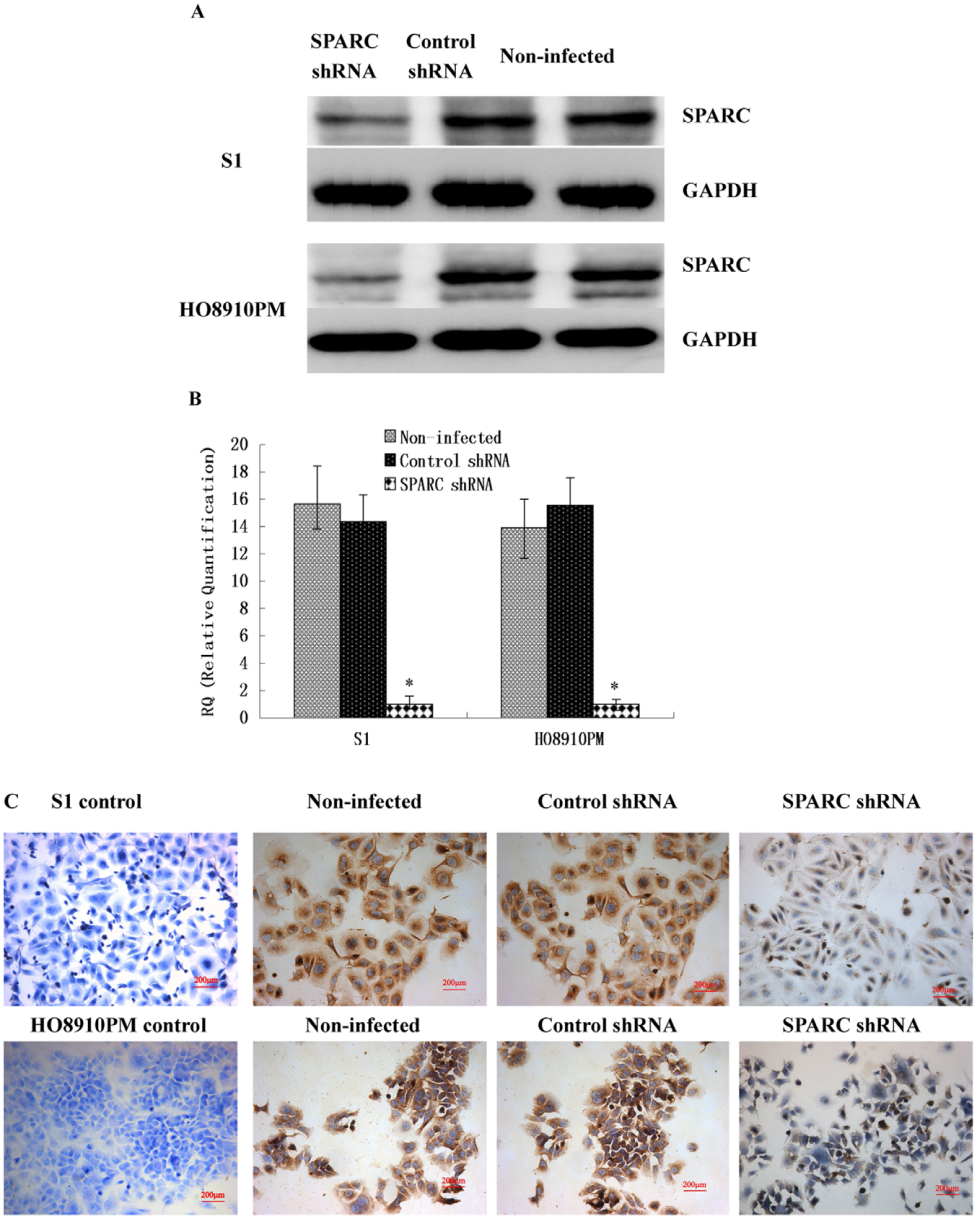
Verification of knockdown of SPARC expression in highly invasive cell line S1 and HO8910PM by lentivirus-mediated RNA interference. (A) SPARC protein expression in lentivirus-infected cells as measured by Western blot. (B) SPARC mRNA expression in lentivirus-infected cells as measured by q-RT-PCR. (C) SPARC protein expression in lentivirus-infected cells as measured by IHC staining (Magnification ×200). *P<0.05 versus control.

### Flow Cytometry Analysis

Cells at log phase were collected and adjusted to a density of 1∼5×10^6^/ml with PBS, and then stained with 20 µl fluorescein isothiocyanate (FITC)-conjugated monoclonal antibodies for 30 min at 25°C. All antibodies including anti-integrin β1, anti-integrin β3 and anti-E-cadherin were purchased from BD Biosciences Pharmingen (San Jose, CA, USA). Finally, the stained cells were analyzed by a flow cytometer. Data analysis was carried out using the program WinMDI. The test was performed in quadruplicates.

### MMPs Activity Assay by Zymography

The matrix metalloproteinases activity assay was performed in 10% SDS-PAGE gel containing 0.1 mg/mL gelatin. 20 µg protein sample was loaded into each lane of SDS-PAGE gel. After electrophoresis, the gel was washed twice with 2.5% Triton X-100 for 1 h at room temperature to remove SDS and incubated at 37°C for over night in reaction buffer (50 mmo1/L Tris–HCl pH 7.4, 8.775 g NaCl, and 1.ll g CaCl_2_). After staining with Coomassie brilliant blue, MMPs activity was identified as clear zones against blue background.

### Statistical Analysis

IHC data were analyzed using chi-square test. The results of cell and molecular biology data were expressed as the mean ± SE. For comparison of means between two groups, a two-tailed t-test was used and for comparison of means among three groups, one-way ANOVA were used. Survival curve was calculated using the Kaplan-Meier method and the log-rank test. Statistical analysis was performed using SPSS software version 13.0. P-value <0.05 was considered statistically significant.

## Results

### Differentially Expressed Genes in the Highly Invasive Subclone S1 and Low Invasive Subclone S21

To discover novel biomarkers for invasive ovarian cancer, we performed microarray analysis to identify the differential gene expression profile of the highly invasive subclone S1 and low invasive subclone S21. These two subclones were derived from the parental SKOV3 human ovarian cancer cell line and had similar genetic backgrounds; therefore, they are suitable for comparative analysis. The microarray data were analyzed using the assigned cut-off expression ratio of 1.5 fold change and *P*-values ≤0.05. The analyses identified 1,596 differentially expressed genes (Table S1 and Figure 1A). Among these genes, SPARC was markedly up-regulated in the highly invasive subclone compared with the low invasive subclone (13.8 fold, *P* = 0.023). Real-time q-RT-PCR, Western blot and IHC results confirmed the overexpression of SPARC in the highly invasive subclone (Figure 1BCD).

**Figure 5:**
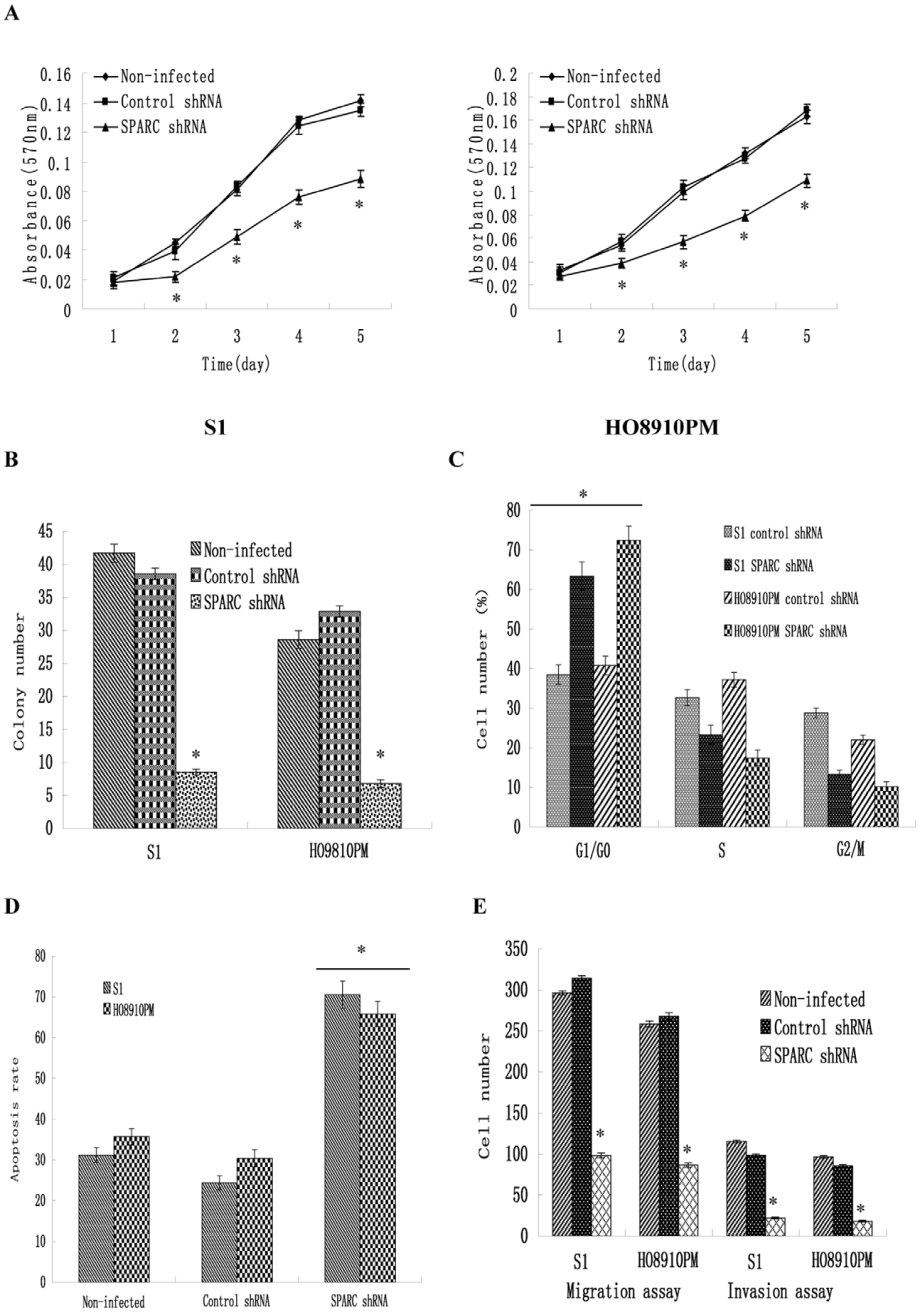
Effect of SPARC knockdown in highly invasive cell line S1 and HO8910PM on cell growth, colony formation, apoptosis, migration and invasion. (A) Cell proliferation as examined by MTT assay. (B) Anchorage independent growth as measured by soft agar colony formation assay. (C) Cell-cycle distributions as measured by flow cytometry. (D) Cell apoptosis as measured by Annexin V-PI assays. (E) Cell migration and invasion assay using Boyden chambers. *P<0.05 versus control.

### Expressions of SPARC in Human Ovarian Tissues

Using both two anti-SPARC antibody in normal ovarian tissues, SPARC expressions were low and mainly focused around the vasculatures in the stroma (Figure 2A and 2F), and in benign ovarian tumors, SPARC expression was also low and mainly focused not only in the stroma but also in the tumor cell cytoplasm (Figure 2B and 2G), finally in most ovarian carcinomas, the immunoreactivity was high, and high SPARC expression was found not only in stroma but also in the cytoplasm of ovarian cancer cells (Figure 2CDE and 2HIJ). Moreover, high SPARC expression was associated with low differentiation, high stage and positive lymph node status of ovarian carcinomas (Table 2 and 3). The results of peptide blocking experiments demonstrated the specificity of the primary antibody (Figure 3ABCDE). To evaluate the prognostic value of SPARC in ovarian cancer, we performed survival analysis using Kaplan-Meier analysis. The result showed that patients with high SPARC expression had a much worse prognosis than those with low SPARC expression (log rank, *p* = 0.004; Figure 3F).

**Figure 6:**
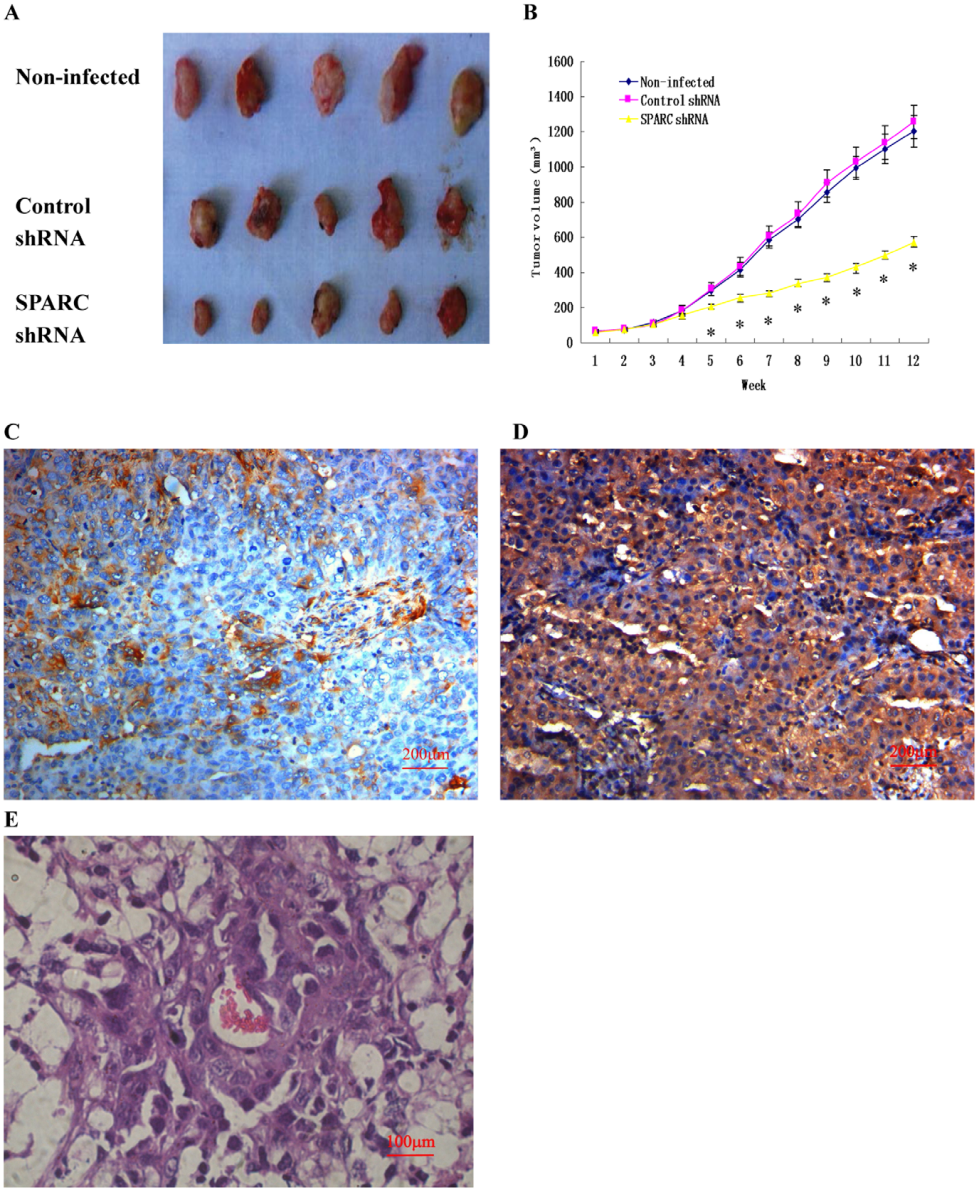
Effect of SPARC knockdown on tumor growth and lung metastasis in nude mice and the downstream target genes of SPARC in ovarian tumor. (A) Photograph of xenografts dissected from nude mice after 3 months subcutaneous inoculation (n = 5). (B) Knockdown of SPARC inhibited tumor growth in vivo. (C) SPARC expression in the tumor formed by SPARC shRNA infected cells as measured by IHC staining (Magnification ×200). (D) SPARC expression in the tumor formed by control shRNA infected cells as measured by IHC staining (Magnification ×200). (E) Photograph of lung metastasis under a microscope after 3 months inoculation through tail vein (n = 10). *P<0.05 versus control.

### Knockdown of SPARC Expression by Lentivirus-mediated RNA Interference

To investigate the role of SPARC in ovarian cancer, we constructed lentivirus vector with SPARC shRNA and infected the highly invasive subclone cells (S1 and HO8910PM). The similar data were achieved in S1 and HO8910PM cells following SPARC shRNA virus infections. After viral infection, more than 80% cells were GFP-positive, indicating a high efficiency of shRNA delivery. Real-time q-RT-PCR, Western Blot and IHC confirmed the down-regulation of SPARC expressions by its shRNA at both mRNA and protein levels, as shown in Figure 4.

**Figure 7:**
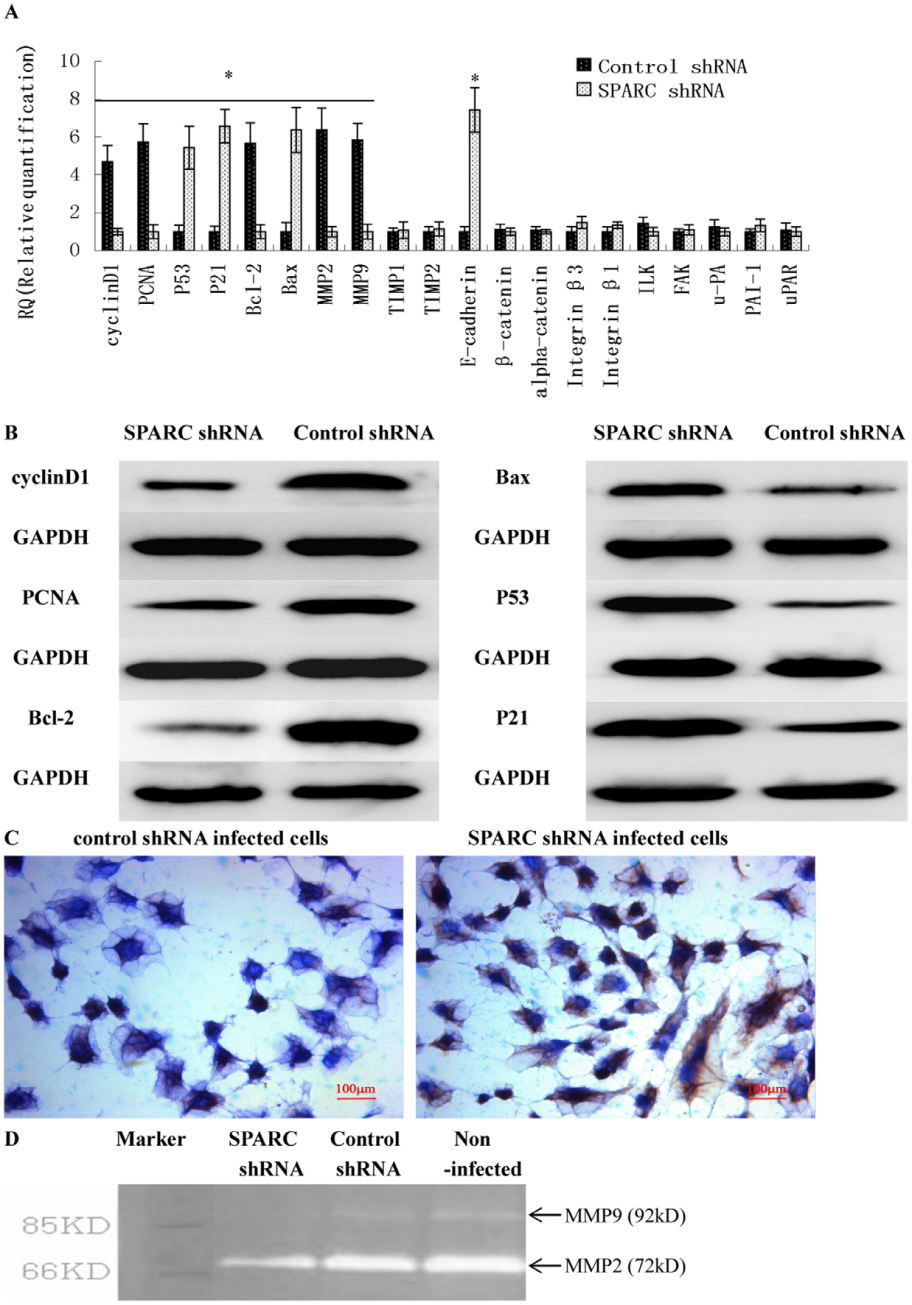
The downstream target genes of SPARC in affecting ovarian cancer cell proliferation, apoptosis and invasion. (A) The downstream target genes of SPARC as measured by q-RT-PCR. (B) P53, P21, Cyclin D1, PCNA, Bcl–2 and Bax protein expression in lentivirus-infected cells as measured by Western blot. (C) E-cardherin protein expression in lentivirus-infected cells as measured by IHC staining (Magnification ×200). (D) MMPs activities measured by zymography. *P<0.05 versus control.

### Knockdown of SPARC Expression Suppressed Ovarian Cancer Cells Proliferation

Next, we investigated the effect of SPARC knockdown on the proliferation of the highly invasive subclone cells (S1 and HO8910PM). MTT results showed that SPARC knockdown significantly reduced cell proliferation of S1 and HO8910PM cells (Figure 5A). In soft agar colony formation assay, S1 and HO8910PM cells infected with SPARC shRNA virus showed significant reduction in the colony formation (Figure 5B). No significant difference was found between control shRNA virus infected cells and non-infected cells.

### Knockdown of SPARC Expression Induced Cell Cycle Arrest at the G1/G0 Phase

To understand the mechanism by which the proliferation of ovarian cancer cells was inhibited by knockdown of SPARC expression, we used flow cytometry to identify the specific phases of cell cycle. As shown in Figure 5C, S1 and HO8910PM cells infected with SPARC shRNA contained 20∼30% more cells at the G1 or G0 (G1/G0) phase (*P*<0.05) compared with the control shRNA infected cells. These data indicated that knockdown of SPARC expression inhibited the proliferation of ovarian cancer cells by blocking their progression from the G1/G0 phase to the S phase during the cell cycle.

**Table 4:** Flow cytometry analysis about E-cadherin, integrin β1 and integrin β3.

	Percent positive cells			*P*
	SPARC shRNA		Control shRNA	
E-cadherin	87.54±6.27	26.73±2.78		<0.01
Integrin β1	98.74±7.83	97.43±8.12		>0.05
Integrin β3	65.54±4.35	63.38±3.97		>0.05

### Knockdown of SPARC Expression Induced Ovarian Cancer Cell Apoptosis

As shown in figure 5D, the percentage of apoptotic cells infected with SPARC shRNA was much higher than that in control shRNA group (P<0.05). No significant difference was found between control shRNA virus infected cells and non-infected cells. These data indicated that knockdown of SPARC expression induced ovarian cancer cell apoptosis.

### Knockdown of SPARC Expression Inhibited Ovarian Cancer Cells Migration and Invasion

We further examined the effect of SPARC knockdown on the migration and invasion of the highly invasive subclone cells (S1 and HO8910PM). As shown in Figure 5E, knockdown of SPARC inhibited ovarian cancer cells invasion and migration. The similar data were achieved in S1 and HO8910PM cells following SPARC shRNA virus infections. The average invading or migrating cell count of SPARC shRNA infected cells was much less than that of control shRNA infected cells. No significant difference was found between control shRNA infected cells and non-infected cells.

### Knockdown of SPARC Expression Inhibited Tumor Growth and Lung Metastasis in Nude Mice

Tumor formation was performed in S1 subclone infected by SPARC shRNA virus in nude mice. Cells were inoculated subcutaneously in nude mice and tumor growth was measured after 3 months. As shown in figure 6A and B, knockdown of SPARC showed a decrease in the size of tumors compare with its control counterpart. There was no significant difference between control shRNA infected cells and non-infected cells in the tumor formation of nude mice. The tumors were dissected, fixed by formalin and embedded by paraffin, then IHC experiments were made, the tumor formed by SPARC shRNA infected cells showed lower SPARC expression (Figure 6C), while the tumor formed by control shRNA infected cells showed higher SPARC expression (Figure 6D). Moreover, figure 6E shows the lung metastasis under a microscope after injection of control shRNA infected cells and non-infected cells through the tail veins of nude mice. About 50% lung metastasis were found after 3 months in the nude mice injected with control shRNA infected cells and non-infected cells, while no lung metastasis was found after injection with SPARC shRNA infected cells in nude mice.

### The Downstream Target Genes of SPARC in Affecting Ovarian Cancer Cell Proliferation, Apoptosis and Invasion

To understand the mechanism of SPARC in ovarian cancer proliferation, apoptosis and invasion, real-time quantitative RT-PCR was used to detect the different expressions of E-cadherin, β-catenin, alpha-catenin, Integrin β3, Integrin β1, ILK, FAK, P53, P21, Cyclin D1, PCNA, Bcl-2, Bax, u-PA, uPAR, PAI-1, MMP2, MMP9, TIMP1 and TIMP2 between SPARC shRNA infected S1 subclone cells and control shRNA infected S1 subclone cells. As shown in Figure 7A, Cyclin D1, PCNA, Bcl–2, MMP2 and MMP9 were significantly down-regulated in SPARC shRNA infected cells. Besides, higher expression levels of E-cadherin, P53, P21 and Bax was found in SPARC shRNA infected cells. There were no significant differences in the expressions of β-catenin, α-catenin, Integrin β3, Integrin β1, ILK, FAK, u-PA, PAI-1, uPAR, TIMP1 and TIMP2 between SPARC shRNA infected cells and control shRNA infected cells. The results of flow cytometry analysis (Table 4) and western blot (Figure 7B) confirmed that E-cadherin P53, P21 and Bax were up-regulated and Cyclin D1, PCNA and Bcl–2 were down-regulated in SPARC shRNA infected cells. IHC experiments revealed that the expression of E-cadherin increased in the cytomembrane of SPARC shRNA infected cells (Figure 7C). Zymography results showed that the MMPs activities were significantly reduced in SPARC shRNA infected cells (Figure 7D).

## Discussion

In this study, using genome-wide transcriptional profiling, we identified that SPARC was overexpressed in the highly invasive SKOV3 subclone compared with the low invasive subclone. Further analysis of SPARC expression in 140 human ovarian tissues revealed that SPARC was overexpressed in malignant ovarian tumors and associated with poor clinicopathologic features. These results suggest that SPARC may play an important role in the development of ovarian cancer. With the antibody AON-5031, similar results were observed in 8 normal ovarian tissues and 24 human invasive ovarian cancers [13], and 14 normal ovarian tissues and 48 ovarian cancers [14]. They all found that SPARC was up-regulated in reactive stroma associated with invasive ovarian cancer. In our study, with the antibody AF941 and bs-1133R, we found that the immunoreactivity of SPARC was heightened not only in stroma but also in the cytoplasm of ovarian cancer cells. In contrast, with the antibody LF-54, another study indicated that SPARC expression is down-regulated in ovarian cancer; exogenous SPARC inhibited the proliferation and induces apoptosis in ovarian cancer cells [21]. But in our study, by lentivirus-mediated RNA interference, we found that knockdown of SPARC expression suppressed cell proliferation, induced cell apoptosis, and inhibited cell invasion and metastasis in ovarian cancer cells. Similar results were also been found in gastric cancer cells [26], glioma cells [27] and melanoma cells [28], by RNA interference. In our research, we found that SPARC was not only secreted by the stroma but also secreted by ovarian cancer cells and may exert important intracellular effects upon these cells. Knockdown of SPARC can inhibit ovarian cancer cell growth and invasion. We found that SPARC diffused from the stroma to cancer domains. In this process, SPARC may play a role in promoting the invasion and metastasis of ovarian cancer. The inconsistent results about the SPARC expression in ovarian tissues may be due to the different SPARC antibodies used in these researches. In conclusion, the functions of SPARC in ovarian cancer need further studies.

In this study, using MTT assay and soft agar colony formation assay, we concluded that knockdown of SPARC suppressed ovarian cancer cell proliferation. Flow cytometer showed that knockdown of SPARC reduced the number of cells in S-phase, while increased the number of cells in G1/G0-phase, indicating G1/G0 arrest. To understand the mechanism of SPARC in ovarian cancer proliferation, we detected the different expressions of Cyclin D1, PCNA, P53 and P21 between SPARC shRNA infected cells and control shRNA infected cells.

Cyclin D1, a member of G1 cyclins, and PCNA (proliferating cell nuclear antigen) are commonly used to assess tumor cell proliferation [29], [30]. P53, a tumor suppressor gene, and P21 (cyclin-dependent kinase inhibitor), a key mediator of p53, can induce cell-cycle arrest in the G1-S checkpoint [31], [32]. In this study, we found that knockdown of SPARC expression could decrease the expressions of Cyclin D1 and PCNA and increase the expressions of P53 and P21, which suggested that depletion of SPARC could inhibit ovarian cancer cell proliferation through activation of a p53/p21^Cip1/Waf1^ pathway dependent to G1-S checkpoint. In melanoma, SPARC also could regulate cell cycle progression and proliferation through the p53/p21 (Cip1/Waf1) pathway [33]. In brief, SPARC could play an important role in ovarian cancer cell proliferation by controlling cell cycle progression, but how depletion of SPARC can lead to activation of the p53/p21 (Cip1/Waf1) signaling pathway needs further study.

Further research showed that knockdown of SPARC induced ovarian cancer cell apoptosis. Apoptosis is modulated partially by Bcl-2 family including apoptosis-inhibiting genes (Bcl-2, Bcl-xL, Mcl-1, A1, Bcl-w) and apoptosis-accelerating genes (Bax, Bak, Bcl-xS, Bim) [34]. In this study, we found that knockdown of SPARC could decrease the expression of apoptosis-inhibiting gene Bcl–2 and increase the expression of apoptosis-accelerating gene Bax, the ratio of Bcl–2/Bax was down regulated. Our result revealed that SPARC could play an effect on apoptosis by changing the ratio of Bcl–2/Bax. Similar results were found in human melanoma, suppression of SPARC in several human melanoma cells triggered apoptotic cell death dependent on p53 and induction of Bax [35]. Programmed cell death, apoptosis is vital for normal development and tissue homeostasis. Our data suggested that SPARC, as an antistress factor, could promote ovarian cancer cell survival through suppression of apoptotic pathways.

Using Boyden chambers and xenografts in nude mice, we concluded that knockdown of SPARC expression inhibited cell invasion and metastasis. This result was opposite to the research using SPARC-null and wild-type mice [36]. By intraperitoneal injection of syngeneic ID8 ovarian cancer cells into SPARC-null and wild-type mice, they found that absence of host-derived SPARC dramatically accelerates ascites formation and peritoneal metastasis in vivo. But recent research found that SPARC-null and wild-type mice were orthotopic injected with ID8 cells, no differences were observed in survival or abdominal lesions between SPARC-null and wild-type mice after OT injection [37]. These results revealed the limitation of using SPARC-null mice to assess the role of SPARC in ovarian cancer progression. So in further researches, we intended to build orthotopic transplantation tumor model in nude mice to perfect our animal experiments.

To clarify the mechanism of SPARC in ovarian cancer invasion and metastasis, after viral infection, we detected the expression of cellular adhesion molecule E-cadherin and integrins, which mediated cell-cell adhesion and cell-extracellular matrix adhesion, and the expression and activity of proteolytic enzymes such as plasminogen activator/plasmin system (uPA-uPAR) and matrix metalloproteinases (MMPs), which degraded the extracellular matrix (ECM). These results revealed that knockdown of SPARC up-regulated the expression of E-cadherin and had no effect on the expression of integrin β1 and integrin β3. SPARC can breakdown cell-cell connections to improve tumor invasion by changing E-cadherin expression. Similar result were also found in melanoma [38], [39], SPARC can down-regulate E-cadherin and stimulate an invasive melanoma phenotype. Another research revealed that exogenous SPARC inhibited αv- and β1- mediated adhesion of ovarian cancer cells to ECM [40]. But in this study, intracellular SPARC did not change the expression of integrin β1 and integrin β3 after viral infection. There must be another pathway about intracellular SPARC promoting cell-extracellular matrix adhesion. Next, knockdown of SPARC decreased the activities of MMP2 and MMP9, but no significant differences were found in the expressions of u-PA, uPAR, PAI-1, TIMP1 and TIMP2 between SPARC shRNA infected cells and control shRNA infected cells. SPARC can make the extracellular matrix degradation by MMPs. Similar results were also found in glioma [41]–[43], SPARC up-regulated the expression of MMP-2 and MMP-9. Our data suggested that depletion of SPARC could promote the homophilic cell-cell adhesion by up-regulating E-cadherin and restrained extracellular matrix degradation by down-regulating MMPs activities to inhibit ovarian cancer cell invasion and metastasis.

In conclusion, overexpression of SPARC is associated with tumor progression of human ovarian cancer. Knockdown of SPARC suppressed ovarian cancer cell proliferation, induced cell apoptosis and inhibited cell invasion and metastasis. All of these informations contribute to a better understanding that intracellular SPARC, as a promoter, improves ovarian cancer cell proliferation, invasion and metastasis.

## Supporting Information

Table S1 **Microarray data.** The differential gene expression profile of the highly invasive subclone S1 and low invasive subclone S21 was identified by microarray analysis.
(XLS)
